# Population Genetic Changes Associated with Long-Term African Swine Fever-Related Management in a Wild Boar (*Sus scrofa*) Population from Northern Hungary

**DOI:** 10.3390/genes17080929

**Published:** 2026-08-08

**Authors:** Lajos Molnár, Viktor Stéger, Petra Zenke

**Affiliations:** 1 Pásztó District Office of the Nógrád County Government Office, Kölcsey u. 35, HU-3060 Pásztó, Hungary; molnar.lajos3@nograd.gov.hu; 2 Department of Genetics and Genomics, Institute of Genetics and Biotechnology, Hungarian University of Agriculture and Life Sciences, HU-2100 Gödöllő, Hungary; steger.viktor@uni-mate.hu; 3 Department of Animal Breeding and Genetics, Institute for Animal Breeding, Nutrition and Laboratory Animal Science, University of Veterinary Medicine Budapest, Istvan u. 2, HU-1078 Budapest, Hungary

**Keywords:** African swine fever, *Sus scrofa*, population management, genetic diversity, microsatellite markers, population structure, wildlife forensic genetics, genetic monitoring

## Abstract

**Background/Objectives:** African swine fever (ASF) has caused substantial wild boar (*Sus scrofa*) mortality across Europe and prompted intensive density-reduction measures, yet its long-term effects on population genetics remain poorly understood. To our knowledge, this is the first study to compare genetic diversity, population structure, demographic signatures, and wildlife forensic identification parameters in the same wild boar population before ASF emergence and after several years of ASF-related management. **Methods:** Wild boar samples collected before ASF emergence in Hungary (*n* = 67) were compared with samples collected after several years of ASF-related management from the same geographic area (*n* = 65). All 132 individuals were genotyped at 13 tetrameric microsatellite loci. Diversity was assessed using allelic richness, allele number, and heterozygosity; differentiation using F-statistics, analysis of molecular variance, and discriminant analysis of principal components; demographic history using a two-phase mutation model; and forensic performance using probability of identity and probability of identity among siblings. **Results:** The post-ASF population showed lower genetic diversity and significant temporal differentiation. Paired Wilcoxon tests indicated a significant reduction in the number of alleles, whereas changes in heterozygosity were not significant. Bottleneck analyses provided no robust evidence of a recent genetic bottleneck. Forensic discrimination power declined modestly while remaining sufficient for individual identification. **Conclusions:** Because no contemporaneous unaffected reference population was available, these changes cannot be attributed specifically to ASF or management rather than to genetic drift or natural turnover, although they are consistent with sustained demographic disturbance. Nevertheless, this study provides the first temporal genetic comparison of a European wild boar population before ASF emergence and after several years of ASF-related management and demonstrates that a routinely used STR marker panel remains suitable for wildlife forensic individual identification despite these genetic changes.

## 1. Introduction

While the epidemiological aspects of ASF have been extensively investigated, relatively few studies have evaluated its population genetic consequences. Although ASF has spread across much of Central and Eastern Europe since its introduction into the European Union in 2014, affecting numerous wild boar populations under long-term disease management, population genetic studies remain scarce. Recent reviews have highlighted that population reduction through hunting, carcass removal, and other management interventions remains a cornerstone of ASF control throughout Europe [1,2]. In Lithuania, Griciuvienė et al. (2022) reported genetic differentiation between ASF-affected and unaffected wild boar populations, suggesting that disease outbreaks and associated population declines may influence population genetic structure [3]. Similarly, Reiner et al. (2021) and Simon et al. (2024) demonstrated the importance of population genetic connectivity for understanding wild boar dispersal in ASF-endangered regions of Germany [4,5]. To date, only a very limited number of studies have examined the genetic consequences of ASF-related population management, despite the widespread occurrence of the disease across Europe. Recent EU-wide surveillance underscores the continued intensification of the epidemic, with notified ASF cases in wild boar in the European Union rising from 7677 in 2024 to 11,036 in 2025 [6]; multi-year surveillance in neighboring Slovakia documents comparable spatio-temporal dynamics of infection and control in free-ranging wild boar [7].

Intensive culling and sustained reductions in population size may decrease effective population size, increase genetic drift, reduce allelic richness and heterozygosity, and alter patterns of gene flow among local populations [8,9]. Such processes may ultimately reduce adaptive potential and affect long-term population viability [10,11]. However, the magnitude and direction of these genetic changes are difficult to predict, as population reduction may be partially counterbalanced by immigration and ongoing gene flow from neighboring populations. Similar genetic effects have been reported in wildlife populations exposed to anthropogenic barriers and habitat fragmentation, where reduced connectivity resulted in measurable genetic differentiation among subpopulations [3].

Because population reduction is considered a key component of ASF control in wild boar populations [12], regions affected by ASF for several consecutive years may experience substantial demographic turnover resulting from the combined effects of disease-associated mortality and intensive management measures. Such demographic changes have the potential to influence the genetic composition of local populations. Recognizing the limited knowledge regarding the long-term ecological consequences of ASF-related interventions, EFSA identified this topic among the priority research areas requiring further investigation [13]. In Hungary, ASF management has included intensive surveillance, diagnostic culling, and compensation schemes coordinated by the National Food Chain Safety Office [14,15].

Microsatellite (short tandem repeat, STR) markers are widely used for assessing genetic diversity, population structure, gene flow, demographic history, and hybridization in wild boar populations [16,17,18,19,20,21,22,23,24]. In addition to ecological applications, STR markers are routinely employed for individual identification and wildlife forensic investigations involving poaching, illegal wildlife trade, hybridization detection, and parentage analyses [25,26,27,28,29]. Their high polymorphism and discriminatory power make them particularly suitable for monitoring temporal changes in population genetic composition. Because the effectiveness of genetic individual identification depends on allele frequencies and genetic variability within a population, temporal changes in genetic diversity may also influence forensic parameters such as the probability of identity (PI) and the discriminatory power of microsatellite marker systems [30].

Unlike previous studies that compared ASF-affected and unaffected wild boar populations, the present study employs a temporal pre- and post-ASF design within the same geographic population. This approach minimizes the influence of spatial genetic structure and provides a rare opportunity to investigate temporal genetic changes associated with prolonged ASF-related demographic disturbance. We therefore aimed to (i) assess temporal changes in genetic diversity, population structure, and allele frequencies following several years of ASF-associated mortality and management; (ii) evaluate whether these changes were accompanied by evidence of a recent genetic bottleneck; and (iii) determine whether temporal shifts in allele frequencies affected the forensic performance of a routinely used STR marker panel for individual identification.

## 2. Materials and Methods

Study area: the study was conducted in Nógrád County, northern Hungary (Figure S1). The study area encompasses the western part of the Mátra Mountains, the Cserhát Hills, and the intervening Zagyva River valley. Wild boar populations in the region regularly move between forested habitats located in mountainous areas and agricultural landscapes situated in the river valley, where food and water resources are more abundant. Seasonal and local movements also occur within the Mátra Mountains and surrounding habitats, potentially contributing to gene flow among neighboring groups. Annual data on wild boar harvest and ASF-related mortality in Nógrád County between 2014 and 2024 were obtained from the Hungarian National Game Management Database (OVA) [31]. A total of 44,684 wild boar were removed from the population in Nógrád County between 2019 and 2024 through harvesting, diagnostic culling, or ASF-related mortality (Table 1). From 2020 onwards, harvest statistics also included animals culled for diagnostic purposes within the ASF surveillance program.

Wild boar muscle samples representing the post-ASF population (*n* = 65) were collected in 2025 from the study area. All samples were obtained from hunted animals submitted by local game managers within the framework of ASF surveillance activities. No live animals were sampled, and no animals were killed specifically for the purposes of this study, in accordance with the relevant veterinary and disease-control regulations and permits.

For temporal comparison, a pre-ASF dataset (*n* = 67) was obtained directly from the nationwide wild boar genetic survey published by Mihalik et al. (2020) [21]. The original dataset consisted of 486 individuals genotyped using the same 13 microsatellite markers applied in the present study. Based on the recorded geographic origin of the samples, only individuals originating from Nógrád County and its immediate surroundings were selected. Consequently, the pre- and post-ASF datasets represented the same geographic area before and after the implementation of ASF-related management measures in Hungary.

Genomic DNA was isolated using a FavorPrep^TM^ Tissue Genomic DNA Extraction Mini Kit (Favorgen Biotech, Ping-Tung, Taiwan) following the provided procedural guidelines. The quality of the extracted DNA was tested using a 1% agarose gel stained with GelRed^TM^ Nucleic Acid Gel Stain (Biotium, Fremont, CA, USA), and the concentration was measured using a Qubit 2.0 Fluorometer (Life Technologies Corporation, Carlsbad, CA, USA). Isolated DNA from the tissue samples was stored at −20 °C until subsequent analysis.

PCR amplification was performed using a multiplexed microsatellite marker set comprising 13 loci described by Lin et al. (2013) [28]. Forward primers carrying universal adapter sequences were fluorescently labeled using the universal primer end-labeling approach described by Blacket et al. (2012) [32], allowing cost-effective multiplex genotyping using four fluorescent dyes. Each 15 μL PCR reaction contained 4 μL of DreamTaq™ Green PCR Master Mix (Thermo Fisher Scientific, Waltham, MA, USA), 0.2 μL of BSA (20 mg/mL; Sigma-Aldrich, St. Louis, MO, USA), 0.5 μM of forward primer, 0.5 μM of unlabeled reverse primer, 5–10 ng template DNA, and PCR-grade water. Cycling consisted of an initial denaturation step at 94 °C for 10 s, followed by 34 cycles of denaturation at 94 °C for 40 s, annealing at 61 °C for 40 s, extension at 72 °C for 50 s, and a final extension step at 72 °C for 20 min.

The amplified fragments were analyzed using an ABI Prism 3500XL Genetic Analyzer using GeneScanTM-500 LIZTM Size Standard (ThermoFisher Scientific, Waltham, MA, USA). During fragment analysis using GeneMapper^®^ ID-X software version 1.4, the minimum detection threshold was set at 150 relative fluorescence units (RFU). To ensure comparability with the pre-ASF dataset published by Mihalik et al. (2020) [21], allele nomenclature and binning of the post-ASF samples were harmonised with the original allele-calling scheme. All electropherograms were manually inspected and allele calls were verified before downstream analyses. Missing genotypes resulted from occasional locus-specific amplification failures rather than sample exclusion. Overall genotype completeness exceeded 94% (overall missing data: 5.2%; pre-ASF: 7.0%; post-ASF: 3.4%). Replicate genotyping was not performed.

### Statistical Analyses

Locus quality was evaluated separately for the pre-ASF and post-ASF datasets by estimating per-locus null allele frequencies using the R package PopGenReport (R version 4.5.1) and by testing for deviations from Hardy–Weinberg equilibrium using the R package pegas. These analyses were performed to identify potential genotyping artefacts, including null alleles, that could influence temporal comparisons. Genetic diversity was evaluated by calculating the number of alleles per locus (Na), observed heterozygosity (HO), and expected heterozygosity (HE) using GenAlEx v6.5 [33]. Allelic richness (AR) was estimated by rarefaction using FSTAT v2.9.4 [34]. To account for differences in sample size and missing genotypes among loci, AR values were standardized to the minimum sample size available across populations. Differences in genetic diversity between the pre-ASF and post-ASF populations were assessed by comparing AR, Na, HO, and HE values. To complement the descriptive comparison of genetic diversity indices, locus-specific values of the number of alleles (Na), observed heterozygosity (Ho), and expected heterozygosity (He) were compared between the pre-ASF and post-ASF populations using paired Wilcoxon signed-rank tests, with loci treated as paired observational units. As a sensitivity analysis, genetic diversity estimates were recalculated after sequential exclusion of the loci exhibiting the greatest temporal differences to evaluate the influence of individual loci on the overall patterns observed.

Genetic differentiation between sampling periods was quantified using the Weir and Cockerham (1984) [35] θ estimator (commonly reported as FST), calculated in GenAlEx v6.5. In addition, analysis of molecular variance (AMOVA) was performed to estimate the corresponding ΦST statistic.

Population structure was investigated using an unsupervised K-means clustering approach implemented in the find.clusters function of the R package adegenet v2.1.11 [36,37]. The Bayesian Information Criterion (BIC) was used to evaluate the most likely number of genetic clusters. The BIC values were inspected to evaluate the number of genetic clusters, and K = 2 was subsequently explored to assess concordance with the temporal sampling groups. Discriminant Analysis of Principal Components (DAPC) was performed using the adegenet package in R. The optimal number of retained principal components (PCs) was determined using the xvalDapc cross-validation procedure. Cross-validation identified 20 retained PCs as providing the highest mean assignment success (Figure S2), and these PCs, explaining 90.6% of the total PCA variance, were retained for the final DAPC analysis.

Recent population bottlenecks were evaluated using BOTTLENECK v1.2.02 [38]. Heterozygosity excess was tested under the two-phase mutation model (TPM) with 95% single-step mutations, 5% multi-step mutations, and a variance of 12, parameters commonly recommended for microsatellite datasets [39]. Statistical significance was assessed using the sign test, the standardized differences test, and the Wilcoxon signed-rank test; the Wilcoxon signed-rank test was given primary weight, as it is considered the most appropriate for datasets comprising fewer than 20 loci.

Contemporary effective population size (Ne) was estimated separately for the pre-ASF and post-ASF populations using the linkage disequilibrium method implemented in NeEstimator v2.1 [40]. Analyses assumed random mating, and Ne estimates were calculated using a critical allele frequency of Pcrit = 0.02, following the commonly recommended setting for microsatellite datasets.

The forensic performance of the microsatellite marker panel was evaluated by calculating the cumulative probability of identity (PI) and the probability of identity among siblings (PIsibs) using GenAlEx v6.5 [33]. PI estimates the probability that two unrelated individuals selected at random from a population share the same multilocus genotype, whereas PIsibs provides a more conservative estimate assuming the individuals are full siblings [30]. Both parameters were calculated separately for the pre-ASF and post-ASF populations using all 13 microsatellite loci.

## 3. Results

### 3.1. Genetic Diversity

A total of 132 wild boar individuals were included in the analyses, comprising 67 pre-ASF and 65 post-ASF samples. Genetic diversity parameters are summarized in Table 2. Overall, the post-ASF population exhibited lower genetic diversity than the pre-ASF population, as reflected by reduced allelic richness, a lower mean number of alleles per locus, and slightly lower observed and expected heterozygosity. Paired Wilcoxon signed-rank tests provided statistical support for a reduction in Na (V = 32, *p* = 0.042), whereas the differences in observed heterozygosity (Ho; V = 46, *p* = 0.610) and expected heterozygosity (He; V = 49, *p* = 0.456) were not statistically significant. The fixation index remained similar between sampling periods, whereas the proportion of polymorphic loci declined in the post-ASF population.

The number of alleles per locus ranged from 2 to 11 in the pre-ASF population and from 1 to 10 in the post-ASF population. The highest levels of genetic diversity were observed at loci 15A and 13E. In the pre-ASF population, expected heterozygosity reached 0.793 at locus 15A and 0.737 at locus 13E, whereas corresponding values in the post-ASF population were 0.719 and 0.609, respectively (Table S1). Quality control analyses showed no evidence of elevated null allele frequencies for loci 11A or 17A, which exhibited the greatest temporal changes in genetic diversity. Per-locus estimates of null allele frequencies and Hardy–Weinberg equilibrium tests are provided in Table S2.

Several loci exhibited reduced diversity in the post-ASF population. The most pronounced reduction was observed at locus 17A, where the number of detected alleles decreased from five to two and expected heterozygosity declined from 0.487 to 0.015. Similarly, expected heterozygosity at locus 11A decreased from 0.481 in the pre-ASF population to 0.045 in the post-ASF population. Reduced allelic diversity was also observed at loci 11B, 13E, and 7B. In contrast, loci 4C, 14A, and 5C showed comparable or slightly higher diversity values in the post-ASF population. Observed heterozygosity generally followed the same pattern as expected heterozygosity (Table S1). Sensitivity analyses showed that sequential exclusion of loci 17A, 13E, and 11A attenuated the magnitude of the observed reduction in genetic diversity; the reduction in the number of alleles persisted, whereas the difference in expected heterozygosity became negligible and reversed once all three loci were removed (Table S3).

### 3.2. Genetic Differentiation

Genetic differentiation between the pre-ASF and post-ASF populations was low but significant according to the Weir and Cockerham estimator (θ = 0.043, *p* = 0.001). Locus-specific differentiation varied among markers, with the highest values observed at loci 11A, 17A, and 13E (Table S4).

**Figure 1:**
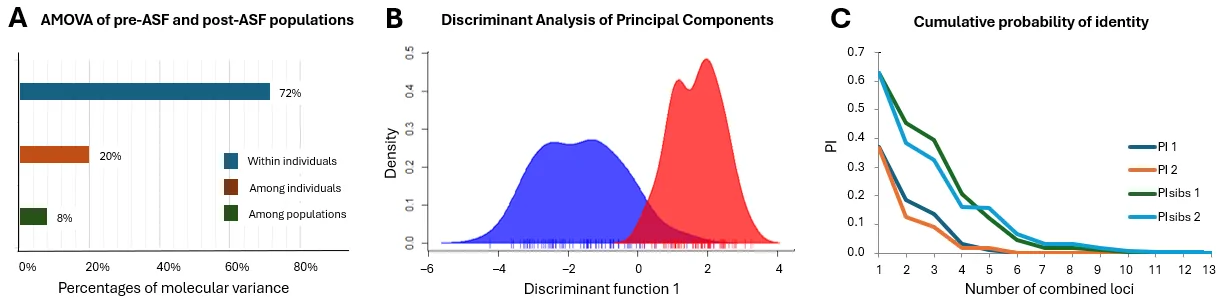
Population genetic differentiation between pre-ASF and post-ASF wild boar populations from northern Hungary. (**A**) Partitioning of molecular variance based on analysis of molecular variance (AMOVA), showing the proportions of genetic variation attributable to differences among populations, among individuals, and within individuals. (**B**) Density distribution of Discriminant Analysis of Principal Components (DAPC) scores for pre-ASF (blue) and post-ASF (red) populations; the analysis was performed using 20 retained principal components. (**C**) Cumulative probability of identity (PI) and probability of identity among siblings (PIsibs) with increasing numbers of microsatellite loci in the pre-ASF (1) and post-ASF (2) populations.

Analysis of molecular variance (AMOVA) further supported the existence of temporal genetic differentiation between populations. Approximately 8% of the total genetic variation was attributable to differences between the pre-ASF and post-ASF populations (ΦST = 0.082, *p* = 0.001), whereas 20% occurred among individuals within populations and 72% within individuals (Figure 1A). These results indicate that most genetic variation remained distributed within populations, although significant temporal differentiation was detectable following several years of ASF-related population management.

### 3.3. Population Structure

Population structure was further investigated using Discriminant Analysis of Principal Components (DAPC). The first discriminant function clearly separated the pre-ASF and post-ASF populations (Figure 1B); the corresponding DAPC density plot is provided in Figure S3). Although some overlap was observed around the centre of the discriminant axis. Similar patterns were obtained when different numbers of principal components were retained, indicating that the observed separation was robust to DAPC parameter settings. These results were consistent with the significant genetic differentiation detected by AMOVA analyses.

To evaluate whether temporal differentiation could also be detected without predefined population labels, an unsupervised K-means clustering analysis was performed. Although the Bayesian Information Criterion (BIC) did not identify a distinct optimal number of genetic clusters (Figure S4), assignment using K = 2 showed 87.9% concordance with the temporal sampling groups, with 52 of 67 pre-ASF individuals and 64 of 65 post-ASF individuals assigned to separate clusters. These findings indicate that the temporal differentiation observed by DAPC was not solely a consequence of predefined group assignment, although the differentiation was not sufficiently strong to produce completely discrete genetic clusters.

### 3.4. Bottleneck

Recent population bottlenecks were evaluated under the two-phase mutation model (TPM). The mean expected heterozygosity of the post-ASF population was 0.373, based on an average of 3.54 alleles per locus. Although the Sign test indicated a marginal deviation from mutation-drift equilibrium (*p* = 0.043), neither the standardized differences test (*p* = 0.081) nor the Wilcoxon signed-rank test (two-tailed *p* = 0.301) detected significant heterozygosity excess. Consequently, the results provided no robust evidence of a recent genetic bottleneck in the post-ASF population under the TPM.

Contemporary effective population size (Ne) estimated using the linkage disequilibrium method was 175.1 for the pre-ASF population and 129.1 for the post-ASF population (Pcrit = 0.02). However, both estimates were associated with wide confidence intervals extending to infinity, indicating limited precision and preventing robust inference regarding temporal changes in effective population size.

### 3.5. Probability of Identity (Pre-ASF vs. Post-ASF)

The discriminatory power of the 13-locus microsatellite panel was evaluated using the probability of identity (PI) and the probability of identity among siblings (PIsibs). Cumulative PI values increased from 4.4 × 10^−7^ in the pre-ASF population to 5.9 × 10^−6^ in the post-ASF population. Similarly, cumulative PIsibs increased from 1.5 × 10^−3^ to 4.4 × 10^−3^ (Figure 1C).

## 4. Discussion

The genetic changes observed in the present study should be interpreted within the broader European context of ASF emergence and control. Following the introduction of ASF virus genotype II into Europe, the disease became established in free-ranging wild boar populations and spread progressively across Central and Eastern Europe, where long-term management has relied primarily on intensive surveillance, carcass removal, and population reduction measures in the absence of an effective vaccine for wild boar [41]. Consequently, many affected populations have experienced prolonged demographic disturbance over multiple years, creating conditions under which gradual changes in genetic composition may occur.

Comparison of samples collected before and after ASF emergence in Hungary revealed reduced genetic diversity and significant temporal genetic differentiation. Formal paired comparisons across loci indicated that only the reduction in the number of alleles (Na) reached statistical significance, whereas changes in heterozygosity were not statistically significant, suggesting that the observed decline in genetic diversity was relatively modest. Although these changes coincided with several years of ASF-associated mortality and population management, the present study cannot distinguish their effects from those of natural demographic processes, such as population turnover or immigration from neighboring populations, because comparable temporal datasets from non-affected populations are not available. Reduced genetic diversity following demographic decline is a well-established consequence of genetic drift and reduced effective population size [8,9]. However, the locus-specific nature of the observed changes suggests shifts in allele frequencies rather than a uniform genome-wide erosion of diversity, which is consistent with the demographic responses expected in highly mobile wildlife species.

The observed temporal differentiation is consistent with the findings of Griciuvienė et al. (2022) [3], who reported genetic differences between ASF-affected and unaffected wild boar populations in Lithuania. In the present study, annual removals remained broadly comparable to those recorded before ASF emergence, but ASF-related mortality and diagnostic culling introduced additional sources of demographic turnover. These findings are consistent with cumulative allele-frequency change over multiple generations, although ongoing gene flow from neighboring areas remain plausible contributors. A complementary pedigree-based analysis of a local pig breed reported a comparable pattern, in which ASF-related culling reshaped population structure while overall genetic diversity remained relatively stable [42].

Despite reduced genetic diversity and significant temporal differentiation, the analyses provided no robust evidence of a recent genetic bottleneck. Similar patterns have been reported in populations undergoing prolonged demographic decline, irrespective of the underlying cause, where bottleneck tests may fail to detect heterozygosity excess despite measurable genetic change [39,43]. A possible explanation for this pattern is that dispersal and gene flow from neighboring populations may have buffered the genetic consequences of local population reductions. Studies of European wild boar populations have demonstrated that landscape connectivity, dispersal, and habitat fragmentation strongly influence population genetic structure [44,45,46,47]. Such processes could maintain gene flow while allowing gradual temporal differentiation to develop, providing one plausible explanation for the absence of a detectable bottleneck in the present study. This possibility is consistent with observations from north-western Italy, where ASF spread has been shown to track wild boar genetic connectivity and admixture zones, indicating that pre-existing population structure can shape epidemic dynamics over short timescales [48]. Although the linkage disequilibrium-based estimate of contemporary effective population size (Ne) was lower in the post-ASF population than in the pre-ASF population, the associated confidence intervals were wide, with infinite upper bounds for both populations. Consequently, the Ne analysis does not provide robust evidence for a temporal reduction in effective population size, which is consistent with the absence of a detectable recent bottleneck.

Changes in genetic diversity may also have practical implications for wildlife forensic applications. Because the discriminatory power of microsatellite marker systems depends on population allele frequencies [30,49], temporal demographic changes can influence forensic identification parameters. In agreement with the observed reduction in genetic diversity, cumulative PI and PIsibs values increased in the post-ASF population. Nevertheless, both parameters remained below the commonly accepted thresholds for reliable wildlife forensic individual identification (PI < 10^−4^; PIsibs < 10^−2^), indicating that the forensic discriminatory power of the marker panel remained only minimally affected despite the observed temporal changes in allele frequencies [30].

Recent reviews of non-human forensic identification emphasize that marker performance is species- and population-specific and must be validated for the target population; this reinforces the need to recalibrate identification parameters such as the probability of identity as population allele frequencies change over time [50,51,52,53].

The marker panel used in this study consisted exclusively of tetrameric STR loci, which generally produce fewer stutter artefacts and facilitate more reliable genotype scoring than dinucleotide repeats [54]. Because the temporal comparison relied on a previously published dataset, technical differences in allele calling cannot be completely excluded. Identical microsatellite loci were analyzed, and allele scoring and binning of the post-ASF samples were harmonised with the original allele-calling scheme of Mihalik et al. (2020) [21]. This harmonisation nonetheless flagged an allele-balance irregularity at locus 17A, and the quality assessment indicated elevated null-allele frequencies at loci 7B and 13E. Because the reduction in diversity and the temporal differentiation were concentrated at loci 17A, 13E, and 11A, the temporal comparison was repeated after sequentially excluding these loci: the reduction in heterozygosity was substantially attenuated, although the overall direction of change remained consistent. This indicates that the overall temporal pattern is not attributable to a single locus, although loci 17A, 13E, and 11A contributed disproportionately to the observed reduction in genetic diversity. Accordingly, the disproportionate contribution of loci 17A, 13E, and 11A should be interpreted with caution. The pronounced changes observed at these loci may reflect stochastic temporal allele-frequency fluctuations, sampling variation, or technical differences between datasets. Because no evidence of elevated null-allele frequencies was detected for loci 11A and 17A, a purely technical explanation appears unlikely for these loci, although it cannot be completely excluded. In contrast, the elevated null-allele frequency detected at locus 13E suggests that technical factors may have contributed to the observed differentiation at this locus. Nevertheless, several limitations should be considered. The study was restricted to a single geographic region and lacked a contemporaneous reference population from a non-ASF-affected area; therefore, the observed temporal changes cannot be attributed exclusively to ASF-related mortality or management and may also reflect natural demographic processes, genetic drift, or immigration. In addition, although the 13-locus microsatellite panel proved sufficient for population genetic analyses and reliable forensic individual identification, microsatellite markers provide substantially lower genomic resolution than genome-wide SNP datasets. SNP-based approaches would offer greater power to estimate effective population size, distinguish drift from gene flow, detect loci potentially affected by selection, and reconstruct demographic history. Finally, although temporal datasets spanning periods before and after ASF emergence remain exceptionally rare, larger sample sizes and additional populations from both affected and unaffected regions will be required to better quantify the long-term genetic consequences of ASF-related population management.

Despite these limitations, the present study provides one of the few temporal population genetic datasets spanning periods before and several years after ASF emergence within the same wild boar population. By combining population genetic analyses with an evaluation of forensic identification performance, it demonstrates that measurable temporal genetic changes can occur while the forensic utility of an established STR marker panel remains largely unaffected. These findings provide a valuable baseline for future genome-wide studies investigating the long-term genetic consequences of ASF-related population management across Europe.

## 5. Conclusions

Prolonged ASF-associated mortality and population management coincided with measurable temporal changes in genetic diversity and population structure in a northern Hungarian wild boar population, while the forensic performance of the microsatellite marker panel remained suitable for individual identification. These findings suggest that temporal demographic changes occurring during prolonged ASF circulation and associated management may influence population genetic composition without producing a detectable bottleneck signature. To our knowledge, this study provides the first temporal genetic comparison of a European wild boar population before ASF emergence and after several years of ASF-related management, while also demonstrating that the forensic performance of a routinely used STR marker panel remains robust despite these genetic changes.

## Figures and Tables

**Table 1:** Annual numbers of harvested wild boar, mortality cases, and total removals recorded in Nógrád County, Hungary, between 2012 and 2024. Total removals represent the sum of harvested animals and mortality cases.

Year	Harvest	Mortality	Total Removal
2012	8432	17	8449
2013	7461	88	7549
2014	9139	59	9198
2015	10,137	24	10,161
2016	8016	20	8036
2017	8636	NA	8636
2018—ASF	9700	156	9856
2019	5490	414	5904
2020	9342	707	10,049
2021	7511	506	8017
2022	7578	167	7745
2023	5652	79	5731
2024	7186	52	7238

**Table 2:** Genetic diversity parameters of a wild boar (*Sus scrofa*) population from northern Hungary before (pre-ASF) and after (post-ASF) the emergence of African swine fever.

Parameter	Pre-ASF (*n* = 67)	Post-ASF (*n* = 65)
Mean number of observed alleles (Na)	4.15	3.54
Mean allelic richness (AR)	3.92	3.3
Mean effective number of alleles (Ae)	2.19	1.92
Mean observed heterozygosity (Ho)	0.414	0.337
Mean expected heterozygosity (He)	0.439	0.372
Mean fixation index (FIS)	0.055	0.052
Effective population size (Ne)	175.1	129.1
Polymorphic loci (%)	100	92.3

## Data Availability

The microsatellite genotype data generated in this study are provided in the Supplementary Materials. The pre-ASF dataset was derived from Mihalik et al. (2020) [21].

## Supplementary Materials

The following supporting information can be downloaded at https://www.mdpi.com/article/10.3390/genes17080929/s1: Figure S1. Study area and sampling location of the wild boar population in Nógrád County. Table S1. Per-locus genetic diversity parameters of the pre-ASF and post-ASF wild boar populations. Table S2. Per-locus quality assessment of the microsatellite markers used in the present study. Figure S2. Cross-validation (xvalDapc) used to determine the optimal number of principal components retained for DAPC. Figure S3. Density distribution of DAPC scores for pre-ASF and post-ASF wild boar populations from northern Hungary. Figure S4. Bayesian Information Criterion (BIC) values obtained from the unsupervised K-means clustering analysis. Table S3. Sensitivity of the temporal diversity comparison to sequential exclusion of the quality-flagged loci (17A, 13E, 11A). Table S4. Per-locus genetic differentiation (Weir–Cockerham FST) between the pre-ASF and post-ASF populations.

## Abbreviations

The following abbreviations are used in this manuscript:

AMOVA	Analysis of Molecular Variance
AR	Allelic Richness
ASF	African Swine Fever
BIC	Bayesian Information Criterion
DAPC	Discriminant Analysis of Principal Components
DNA	Deoxyribonucleic Acid
F	Fixation Index
FST	Fixation Index (Weir and Cockerham estimator, θ)
HWE	Hardy–Weinberg Equilibrium
He	Expected Heterozygosity
Ho	Observed Heterozygosity
Na	Number of Alleles
Ne	Effective Number of Alleles
OVA	Hungarian National Game Management Database
PCR	Polymerase Chain Reaction
PI	Probability of Identity
PIsibs	Probability of Identity among Siblings
RFU	Relative Fluorescence Unit
SNP	Single Nucleotide Polymorphism
STR	Short Tandem Repeat
TPM	Two-Phase Mutation Model
ΦST	AMOVA-based Fixation Index (Phi-statistic)
