# Supplementary material for: Population Genetic Changes Associated with Long-Term African Swine Fever-Related Management in a Wild Boar (Sus scrofa) Population from Northern Hungary
Source: Genes (Basel). 2026 Aug 8;17(8):929. doi: 10.3390/genes17080929 (PMC13512481; doi:10.3390/genes17080929)
Supplement: Supplementary file 1 [file genes-17-00929-s001.zip › genes-4440238-supplementary.pdf]

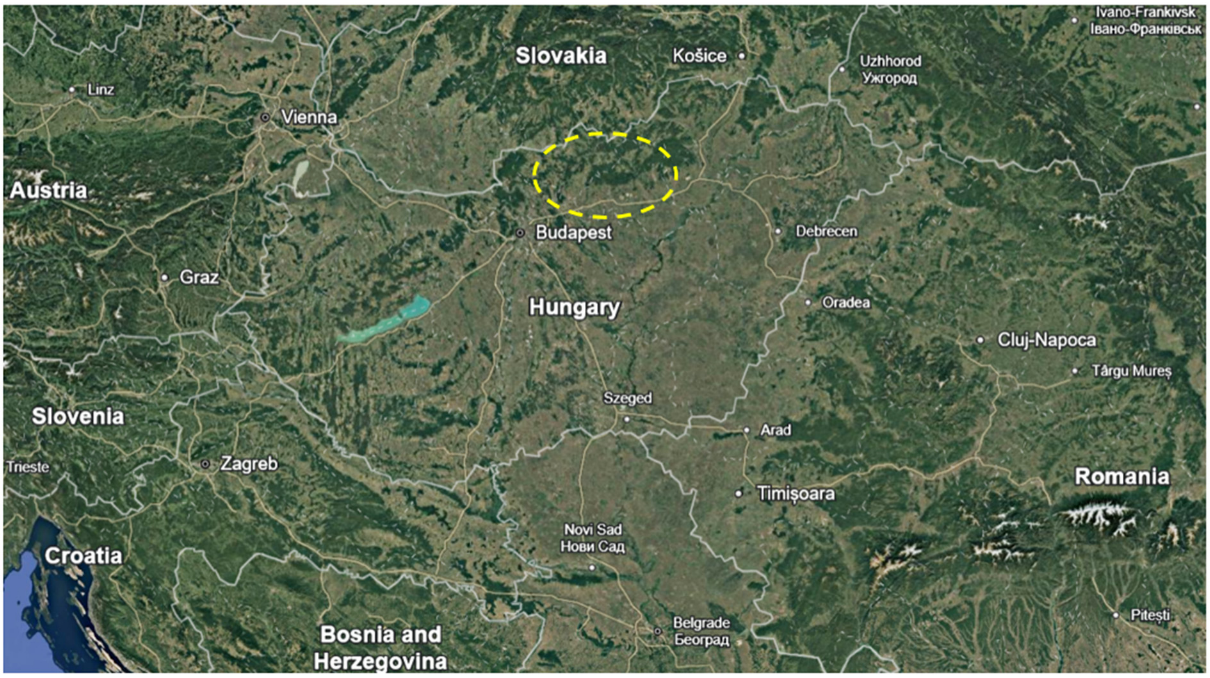

Figure S1. Study area and sampling location of the wild boar population in Nógrád County, northern Hungary. The dashed rectangle indicates the geographic area from which both the pre-ASF and post-ASF samples originated. Sampling locations of the 65 post-ASF individuals analyzed in the present study (34 males and 31 females), collected in 2025–2026 following several years of African swine fever (ASF)-related management, are shown.

Table S1. Per-locus genetic diversity parameters of the 13 microsatellite markers in the pre-ASF and post-ASF wild boar (*Sus scrofa*) populations from northern Hungary. Na = number of alleles; Ho = observed heterozygosity; He = expected heterozygosity; pre-ASF = samples collected before the emergence of African swine fever; post-ASF = samples collected after several years of ASF-related population management.

Locus	Na pre-ASF	Ho pre-ASF	He pre-ASF	Na post-ASF	Ho post-ASF	He post-ASF
4B	3	0.41	0.43	3	0.45	0.43
11B	5	0.20	0.30	4	0.45	0.45
1A	3	0.09	0.14	2	0.18	0.17
4C	4	0.60	0.56	5	0.57	0.61
17A	5	0.61	0.49	2	0.02	0.02
15A	11	0.72	0.79	10	0.59	0.72
13E	7	0.58	0.74	6	0.42	0.61
14B	2	0.04	0.04	2	0.02	0.02
14A	2	0.48	0.43	2	0.52	0.50
5C	3	0.61	0.62	3	0.69	0.66
7B	5	0.42	0.66	4	0.42	0.60
11A	2	0.59	0.48	2	0.05	0.05
1B	2	0.02	0.02	1	0.00	0.00

Table S2. Per-locus quality assessment of the 13 microsatellite markers used for temporal comparison of the pre-ASF and post-ASF wild boar (*Sus scrofa*) populations. Null allele frequencies were estimated using the R package *PopGenReport,* and deviations from Hardy–Weinberg equilibrium (HWE) were evaluated using exact tests implemented in the R package *pegas*. Negative estimates of null allele frequency were set to zero because they indicate no evidence of null alleles.

**Locus**	**Null allele frequency (Pre-ASF)**	**HWE exact *p* (Pre-ASF)**	**Null allele frequency (Post-ASF)**	**HWE exact *p* (Post-ASF)**
1A	0.216	0.0359	0.000	1.0000
1B	0.000	1.0000	NA	1.0000
4B	0.018	0.1673	0.000	0.5992
4C	0.000	0.0527	0.033	0.9187
5C	0.012	0.6921	0.000	0.3650
7B	0.214	<0.0001	0.184	0.0004
11A	**0.000**	0.0792	**0.000**	1.0000
11B	0.194	0.0046	0.002	0.4059
13E	0.118	0.0011	0.182	0.0023
14A	0.000	0.4053	0.000	0.8124
14B	0.000	1.0000	0.000	1.0000
15A	0.046	0.0008	0.095	0.0275
17A	**0.000**	0.0424	**0.000**	1.0000

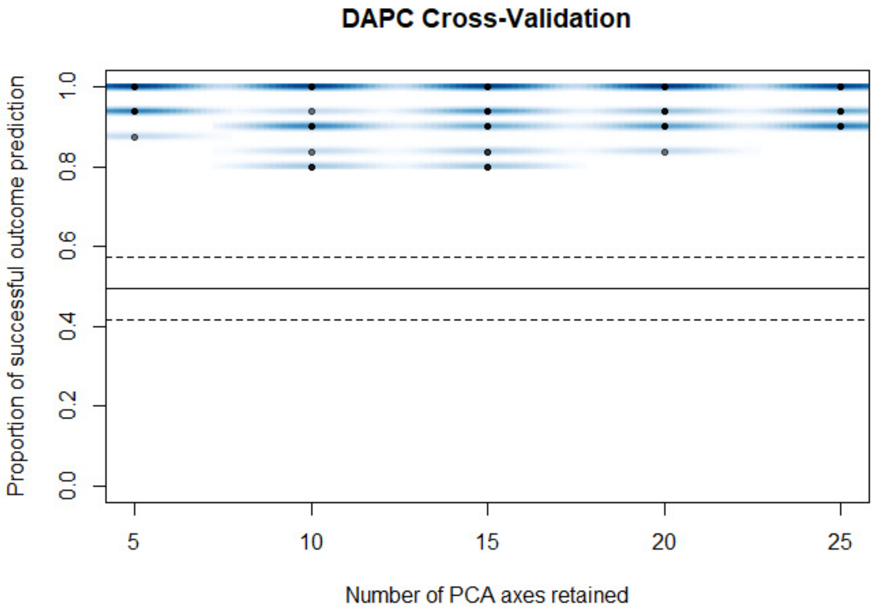

Figure S2. Cross-validation (xvalDapc) used to determine the optimal number of principal components retained for Discriminant Analysis of Principal Components (DAPC). Assignment success remained consistently high across the evaluated range of retained principal components (5–25), with the highest mean assignment success obtained using 20 retained principal components. These PCs explained 90.6% of the total PCA variance and were subsequently used for the final DAPC analysis.

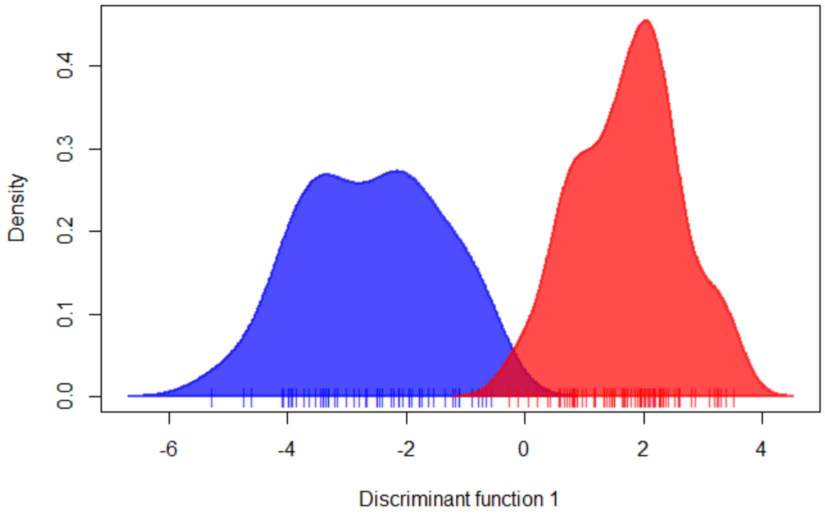

Figure S3. Density distribution of Discriminant Analysis of Principal Components (DAPC) scores for pre-ASF (blue) and post-ASF (red) wild boar populations from northern Hungary. DAPC was performed using the optimal number of principal components identified by cross-validation (20 PCs). The first discriminant function revealed clear temporal genetic differentiation between the two sampling periods, although limited overlap was observed between the populations.

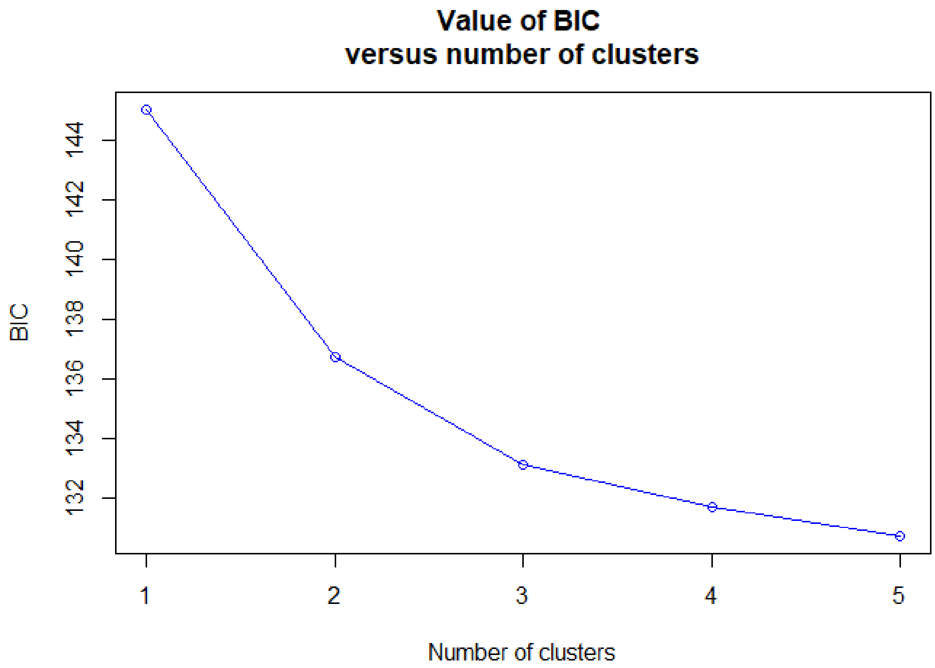

Figure S4. Bayesian Information Criterion (BIC) values obtained from the unsupervised K-means clustering analysis (*find.clusters*) of 132 wild boar individuals. The BIC decreased gradually with increasing numbers of clusters and did not indicate a distinct optimal number of genetic clusters.

Table S3. Sensitivity of the temporal genetic-diversity comparison to sequential exclusion of the quality-flagged loci (17A, 13E, 11A). N_{loci} = number of loci retained; Na = mean number of alleles per locus; H_e = mean expected heterozygosity (biased estimator, GenAlEx convention). H_e change is the relative difference from the pre-ASF to the post-ASF sample (negative = reduction).

**Loci retained**	N_{loci}	**Na pre**	**Na post**	H_e **pre**	H_e **post**	H_e **change**
All loci	13	4.15	3.54	0.439	0.370	-15.6%
Excluding 17A	12	4.08	3.67	0.435	0.400	-8.0%
Excluding 17A, 13E	11	3.82	3.45	0.407	0.381	-6.5%
Excluding 17A, 13E, 11A	10	4.00	3.60	0.400	0.415	+3.6%

Removing locus 17A alone approximately halves the apparent reduction in expected heterozygosity (from -15.6% to -8.0%), and removing all three quality-flagged loci reverses the direction of change. This indicates that the overall reduction in genetic diversity is concentrated at, and largely driven by, these three loci. The result is consistent with a genuine but locus-specific signal; however, because these same loci carry the principal quality-control caveats, confirmation of the locus-specific changes from the original electropherograms is recommended.

Table S4. Per-locus genetic differentiation between the pre-ASF and post-ASF wild boar (*Sus scrofa*) populations from northern Hungary, expressed as the Weir–Cockerham F_{ST} (θ) estimator calculated in GenAlEx v6.5 (999 permutations). Loci are listed in the order analyzed. The overall value corresponds to the multilocus θ reported in the main text.

**Locus**	**4B**	**11B**	**1A**	**4C**	**17A**	**15A**	**13E**	**14B**	**14A**	**5C**	**7B**	**11A**	**1B**	**Overall (θ)**
**FST**	0.005	0.019	0.001	0.016	**0.126**	0.014	**0.097**	0.004	0.034	0.006	0.005	**0.225**	0.005	**0.043**

**Locus**	**4B**	**11B**	**1A**	**4C**	**17A**	**15A**	**13E**	**14B**	**14A**	**5C**	**7B**	**11A**	**1B**	**Overall (θ)**
**FST**	0.005	0.019	0.001	0.016	**0.126**	0.014	**0.097**	0.004	0.034	0.006	0.005	**0.225**	0.005	**0.043**

The three highest values (11A, 17A, 13E) coincide with the loci flagged in quality control: 17A showed an allele-balance irregularity during binning harmonisation, 13E showed elevated null-allele frequencies (Table S2), and 11A showed the strongest per-locus differentiation together with a near-complete loss of observed heterozygosity in the post-ASF sample.
